# Supplementary figures and images for: Pathway-targeting gene matrix for Drosophila gene set enrichment analysis
Source: PLoS One. 2021 Oct 28;16(10):e0259201. doi: 10.1371/journal.pone.0259201 (PMC8553153; doi:10.1371/journal.pone.0259201)

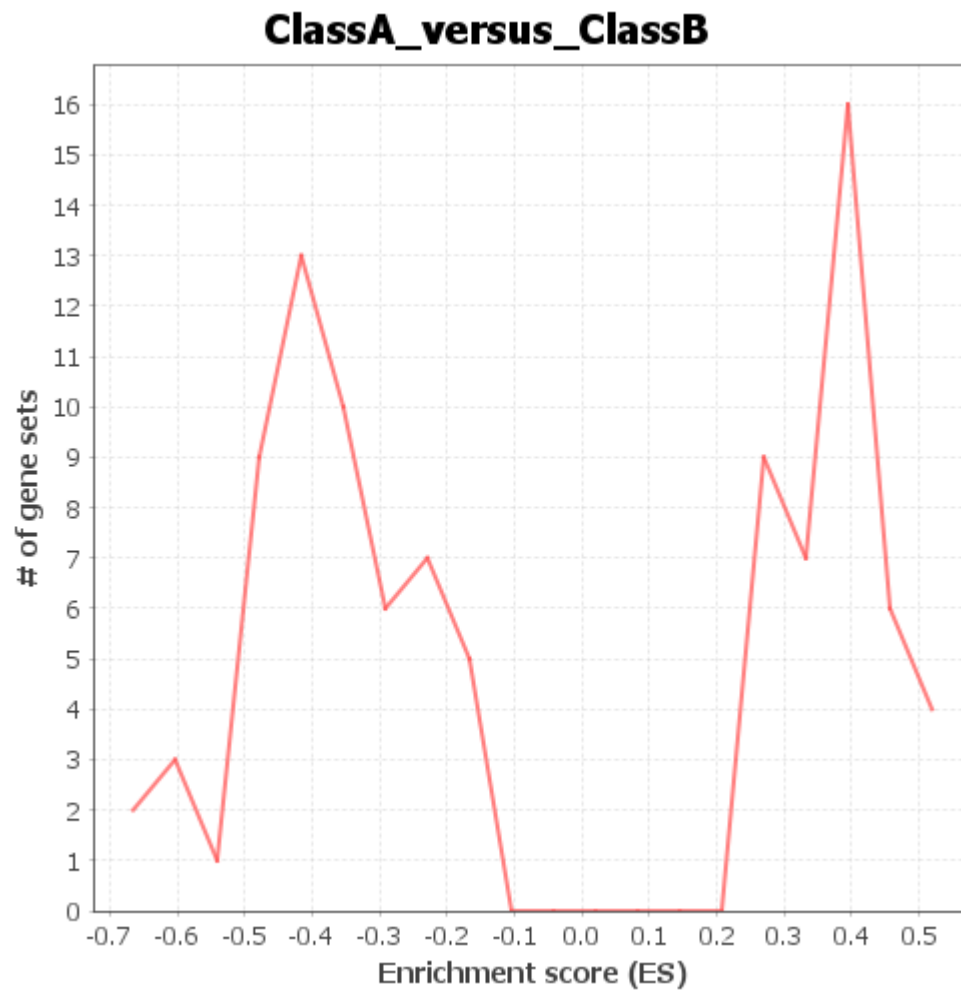

Supplement: S1 Fig — (PDF) [file pone.0259201.s009.pdf]
